# Environmental risk management: A participatory diagnosis from a rural school in Colombia

**DOI:** 10.4102/jamba.v15i1.1510

**Published:** 2023-11-27

**Authors:** Carolina Moncayo, Christian Benitez, Víctor Quintero, Carolina González, Jorge Muñoz, Claudia Hernandez, Manuel Benavides

**Affiliations:** 1 Department of Social Communication, School of Law, Political and Social Sciences, University of Cauca, Popayán, Colombia; 2 Wireless and Radio Research Group, Department of Telecommunications, School of Electronics and Telecommunications Engineering, University of Cauca, Popayán, Colombia; 3 Computational Intelligence Research Group, School of Electronics and Telecommunications Engineering, University of Cauca, Popayán, Colombia

**Keywords:** environmental risk management, participatory diagnosis, rural school, prevention of environmental risk, communication processes

## Abstract

The rural population is potentially exposed to several environmental risks. Environmental risk management is a recurring challenge in rural educational communities that are far from large urban areas. Assessing and identifying the knowledge on prevention and response to environmental risks and natural disasters is fundamental for planning and designing educational programs for communities to face disasters. This article presented the practical application of a participatory methodology to obtain information about the knowledge and experience in the prevention and attention of environmental threats and natural disasters in the rural school Las Huacas, village of Quintana, municipality of Popayán, in the region of Cauca, Colombia. The diagnosis methodology consisted of three phases: preparatory, fieldwork and analytical. The participatory diagnosis allowed the construction of instruments and tools for social interaction, the conduction of dynamic workshops and the systematisation of the collected information. The results evidenced the lack of knowledge on environmental disasters and how parents and children must face them. The absence of communication facilities implied a high degree of vulnerability, which was compensated with the awareness, commitment and participation in the processes led by the educational community. The next challenge is the participatory construction of a scholar environmental risk-management plan supported on appropriated information and communication technologies.

**Contribution:**

This article presents the practical application of a participatory methodology to obtain information about the rural context and the knowledge and experience in the prevention and attention of environmental threats and natural disasters in a rural school in Cauca, Colombia.

## Introduction

The effects of climate change on the planet are becoming increasingly evident in cities and rural areas where it is now common to see disasters such as forest fires, droughts, hailstorms, floods and landslides, among others. Before the coronavirus disease 2019 (COVID-19) pandemic, according to the sustainable development goals report in 2020, 39 million people in the world were affected by different environmental events or disasters (United Nations [UN] 2020) and in the region of Cauca, Colombia, 113 deaths were reported because of natural disasters and 192 062 families were affected between 2008 and 2018, highlighting the fact that hydrometeorological hazards affect 59.7% of the population of the region (United Nations Development Program [UNDP] 2019). In this sense, the importance of being aware, prepared and ready to act in case of natural disasters is relevant for all communities, especially for rural communities and educational environments, in which fathers, mothers, children and teachers are found.

Colombia, because of its geographic position and climatic conditions, has a great diversity and richness of flora and fauna that constantly faces different environmental challenges, many of which are associated with human intervention on its territory. In Colombia, there are different events such as earthquakes, floods, droughts, landslides, avalanches, fires and epidemics, among others, which are registered and reported by the National Unit for Disaster Risk Management (UNGRD) of Colombia (UNGRD 2012). In sparsely populated rural areas, there are problems associated with basic service infrastructure, security, development opportunities, extreme poverty and high deforestation. According to the Colombian Government’s characterisation of deforestation (Ministry of Environment and Sustainable Development [Minambiente] of Colombia 2016), one of the factors that most contribute to environmental degradation is human activities: expansion of the agricultural frontier for extensive cattle ranching, planting of illicit crops, logging, illegal mining and population growth, among others. Between 2000 and 2019, the country registered a loss of nearly 2.8 Mha of forest (Planning National Department [DNP] of Colombia 2020).

According to the Institute of Hydrology, Meteorology and Environmental Studies (IDEAM) of Colombia, Colombia in 2012 had 59 924 000 ha of forests representing 52.5% of the continental territory, which contains high biodiversity; unfortunately, between 1990 and 2010, the country lost 6.2 Mha, equivalent to a deforestation rate of 310 349 ha/year. In the Cauca region, the deforestation rate for the 2017–2018 period corresponded to 0.9% (IDEAM 2018). The above fact has motivated different national organisations and institutions such as IDEAM, the UNDP, the United Nations Programme on Reducing Emissions from Deforestation and Forest Degradation (UN-REDD), the Food and Agriculture Organization of the United Nations (FAO) and the Ministry of Environment and Sustainable Development of Colombia itself in coordination with territorial entities to work proactively on mitigating the environmental, social and economic impacts of deforestation. Thus, one of the focuses prioritised by the Colombian State to meet this objective is an awareness raising and educational training in environmental risk management, especially for children in vulnerable conditions, focusing intervention strategies and mechanisms towards rural communities. However, although progress has been made in the quality and coverage of education in Colombia (Organisation for Economic Co-operation and Development [OECD] 2016), more and better opportunities focused on education on environmental risk management in rural areas are required for increasing the knowledge about potential environmental risks to enhance the community capabilities in the analysis of risk and management of environmental hazards.

Based on the earlier discussed points, the research project: ‘Use and Appropriation of Information and Communication Technologies for Risk Management in the Rural Environment: A Response to the Challenge of Climate Change from the School’, focuses on one of the priority situations defined by the United Nations Children’s Fund (UNICEF): Risk Management, understood as a right and a priority for disaster risk reduction focused on children (UNICEF 2016). United Nations Development Program defines risk management as:

[*A*] complex social process whose ultimate goal is the reduction or the permanent prevention and control of disaster risk in society, in line with and integrated to the achievement of sustainable human, economic, environmental and territorial development patterns. (UNDP 2003:30)

Likewise it states that the frequency and dispersion of natural disasters and the growth of the child population cause the number of children at risk to increase, which makes it necessary to promote risk management through the consolidation in a participatory manner of mechanisms that decrease the negative consequences in the face of possible disasters (UNICEF 2016). In this sense, this article is the result of a participatory diagnosis on the knowledge and management in the prevention and attention to environmental threats and natural disasters in the rural school Las Huacas, village of Quintana, municipality of Popayán, in the region of Cauca, Colombia. This article is organised as follows: Section 2 describes the context of the study population; Section 3 provides a perspective on environmental risk management from a rural perspective; Section 4 introduces the methodology applied in the participatory approach; Section 5 includes the results and their analysis and finally, Section 6 includes the conclusions of the diagnosis.

## Study context

The educational community of the school Las Huacas is a rural community of great importance for food security and environmental sustainability in the municipality of Popayán, Cauca, Colombia. The geographic, demographic, historical and environmental conditions are described further in the text.

### Geographic context

The rural school Las Huacas is located in the township of Quintana, in southwestern Colombia. The township of Quintana is made up of the following villages: Quintana, El Canelo, San Juan and San Ignacio (Pan American Health Organization [PAHO], Sustainable Development Goals Fund [SDGF] & Asociación De Cabildos Genaro-Sánchez 2010) and is located 24 km east of Popayán, on the micro-watershed of the Las Piedras River. The road that connects Popayán with the township is unpaved and mountainous; it is a lightly travelled road and most vehicles or means of transport found in the area are motorcycles and horses.

Much of the township of Quintana is located at an altitude of 2500 m above sea level, its climate is generally cold and in the highest parts of the region, there are paramos and nature reserves (Territorial Development Financial Institution [FINDETER] of Colombia & Inter-American Development Bank [IDB] 2017). The main water stream is the Las Piedras River, which supplies the main aqueduct for the city of Popayán. The main crops found in the area are corn, vegetables, beans, fruit trees, coffee, sugarcane, banana, fique, mulberry, stubble and blackberry (PAHO et al. 2010).

### Demographic context

There are two indigenous reservations in the municipality of Popayán: Poblazón and Quintana, which are part of the Association of Indigenous Councils of the Central Zone Genaro Sánchez (*Asociación de Cabildos de la zona Centro Genaro Sánchez*) and the Regional Indigenous Council of Cauca (CRIC, *Consejo Regional Indígena del Cauca*). The Quintana indigenous reservation is located within the township of the same name. In this same township, there is also a peasant population organised by the Peasant Association of Popayán (ASOCAMPO, *Asociación Campesina de Popayán*) and the Peasant Association of the Village of Quintana (ASOPROQUINTANA, *Asociación Campesina de la Vereda de Quintana*) (PAHO et al. 2010). By 2021, the number of inhabitants of the township of Quintana was expected to reach 958 inhabitants (Popayán Municipal Mayor’s Office 2016). Its inhabitants have found agrotourism as an alternative to support the environmental conservation process and its agricultural activities such as organic fruit, vegetables, dairy cattle, sheep and trout farming.

### Historic context

The indigenous and peasant communities of the township of Quintana have managed to coexist over the years, but their history has been marked by armed struggles, state abandonment and land dispossession, among others. The onslaught of violence by the landowners of the time and illegal groups where the armed struggle was superimposed on the social conflict, generated violence and death for the same settlers (Peñaranda Supelano 2015). One of the most visible armed groups in the region was the Quintín Lame Armed Movement, founded in honour of the indigenous leader Manuel Quintín Lame (1880–1967), who led an armed struggle to change the power model and ‘counteract’ violence towards the end of the 20th century. It should be noted that other armed groups such as the Revolutionary Armed Forces of Colombia (FARC, *Fuerzas Armadas Revolucionarias de Colombia*), M-19 and the National Liberation Army (ELN, *Ejército de Liberación Nacional*) were also present in the region. Peace negotiations led to the demobilisation of some of these groups (Peñaranda Supelano 2015).

### Environment context

The main environmental problems revolve around the Las Piedras River. In some of the villages, there is a predominant and increasing deficit of forest cover, and it is worth noting that: external actors cut down trees near water sources; the forests are used for firewood extraction and construction of native houses; deforestation is uncontrolled and without reforestation plans and land is also burnt because of agricultural and social practices, including the lack of adequate waste management and protection of water resources. The area’s hamlets are located on the slopes of the Puracé volcano, which makes the inhabitants of this geographic area highly vulnerable to different natural phenomena, such as landslides that occur in some sectors of the township, which intensify during the rainy season. Erosion is categorised as moderate to severe because of the absence of trees and the irregularities of the relief (PAHO et al. 2010).

## Perspective of environmental risk management in rural areas

### Problem identification

Based on the context of the village of Quintana, it is important to highlight the lack of communication channels, which prevents a timely strategy for risk prevention and disaster response, implying a high degree of vulnerability of the population. In this sense, the importance of the use of Information and Communication Technologies (ICT) to establish communication between the school, parents and the community in general was analysed, allowing for a reduction in the degree of vulnerability.

### Development and risk management

The concept of development is diverse, dynamic and polysemic and has not been free of discussions and controversies over time, which is why it cannot be defined universally, but rather contextually. One of the concepts of alternative development was born in the 1980s, which is accompanied by various adjectives such as human development, local development and sustainable development (Becerra Lois & Pino Alonso 2005). Thus, the human-scale development proposed by Max-Neef, Elizalde and Hopenhayn (1989) is relevant. The central element of his conception is based on evaluating the world, people and especially their processes from a transdisciplinary perspective. Therefore, this conception of development is relevant in this research proposal, which is related to a process of exchange between various disciplines being the focus of attention of the educational community of the rural school Las Huacas and the need to minimise risks and be prepared for any emergency, as well as to generate conditions that allow the access to communication processes within the municipality. In this sense, the research project is developed from the perspective of local development, focusing on human, cultural and environmental aspects (Max-Neef et al. 1989). Local development should be understood as a process led by the community, organised in a defined territory, where local resources actively participate: human, material, natural, financial and social, for the improvement of their living conditions (Carvajal Burbano 2011). For the present study, local development is understood from the school’s own needs, not only of the entities that comprise it but also of the geographical context in which it is located, in addition to the participation of the actors that are part of their autonomy process, including the change of behaviours associated with generating greater security in the face of natural phenomena and climate change.

## Research methods and design

The methodology used in this research was participatory, from the community, based on its interest and active contribution, given that the community is the protagonist of the diagnosis (Rodríguez Gómez, Gil Flores & García Jiménez 1999). In this sense, the methodology provides a qualitative approach, which makes it possible to enter into the experience and previous knowledge of the individuals, implying the understanding of the context and the reality lived by them. In this way, it is possible to clarify and diagnose the needs, strengths and knowledge of the educational community regarding environmental risk management. The methodological process was established in three phases: preparatory, fieldwork and analytical.

Preparatory: a review of established laws, projects and plans involving the educational community in environmental risk management from a school and rural setting was conducted, which allowed the construction of instruments and tools for social interaction with the community and the collection of information (Montemurro & Opazo Bunster 2006; Pérez Fernández, Sáenz Gómez & Gómez Vega 2016; Valencia Duque & Hernández 2019). From the preparation of the social interaction with the community and as a part of the participatory diagnosis, the facilitating team looks for the tools and the ways to get information from the community about student context, knowledge of environmental risks and communication processes.

Fieldwork: it was developed from an ethnographic and qualitative approach, conducting participatory, playful and dynamic workshops, to know in detail the knowledge of teachers, parents, children and the needs in general regarding environmental risk management and its relationship with the educational, environmental and social context.

Analytical: the information collected was systematised, and a participatory and descriptive diagnosis was generated regarding the knowledge of environmental problems and environmental risk management, to conclude how to implement and develop the educational-communicative workshops regarding the problems and needs of the community. It is important to highlight that, within this phase, the following categories of analysis were identified: student context, knowledge of risks and communicational processes. In the first category, student context, the family nuclei, characteristics of the region, daily activities, jobs of fathers, mothers and guardians and characteristics of the population were identified. In the second category, knowledge of risks, previous knowledge on environmental risk management, disasters and natural phenomena were evidenced. The third category, communication processes, made it possible to understand what means of communication exist in the area, what communication channels are known and used by children, fathers, mothers, guardians and teachers, which are used for internal and external communication in the school, as well as to understand the functions of the media.

### Ethical considerations

Ethical clearance to conduct this study was obtained from the University of the Cauca, Ethics Committee for Scientific Research (No. VRI4932). The people who participated in the participatory diagnosis were adults and their children. The adults signed a written form authorising their participation, and they also signed a written form authorising their children too. To maintain the confidentiality of data, the names of the people who participated in the study are not indicated in the article.

## Results and analysis

### Findings and achievements

The social contributions arising from the research are framed in the above categories of analysis, which are addressed from the results obtained from the fieldwork with children, parents, guardians and teachers.

### Regarding the student context

The rural school Las Huacas at the village of Quintana is made up of 16 students (seven boys and nine girls), a teacher and a food handler. The boys and girls are between the ages of 5 and 11 years old, attending grades transition to fifth grade of primary school, in a multigrade modality. Of the 16 students, nine (56.2%) live in the village of Quintana, four (25%) in the village of El Canelo, one (6.2%) lives in the village of San Ignacio and two (12.6%) do not register their place of residence. A quarter of the children are in fifth grade.

**FIGURE 1:**
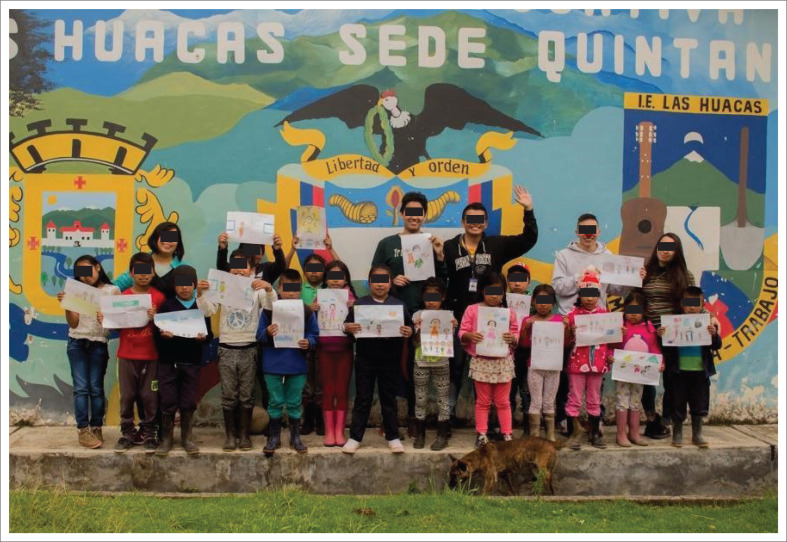
Work team with the school members. Children with their drawings.

**FIGURE 2:**
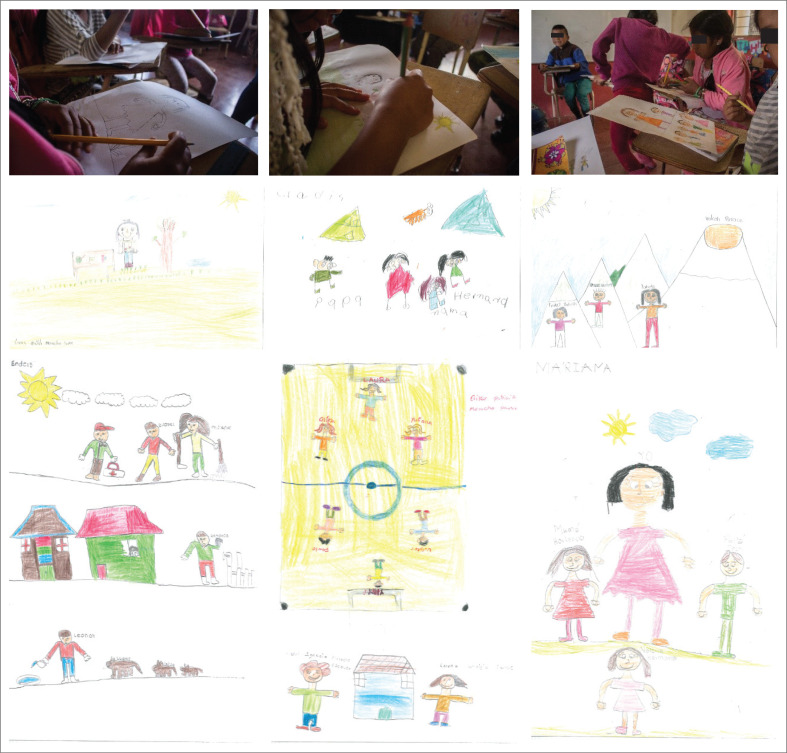
Children drawing and some of their paintings.

The children highlight important aspects about their families, context, interests and their relationship with their environment. The children’s families are characterised as nuclear and extended. Of the total number of students, six (37.5%) affirm that they are part of a nuclear family, that is, made up of parents and children; two (12.6%) do not live with their biological father, but with their stepfather; three (18.8%) live in an extended family, that is, a family also made up of cousins, uncles, aunts, grandparents, grandmothers, nephews, nieces, etc.; six (37.5%) do not show the relationship they have with the people they live with, so it is not possible to determine what type of family they have and one (6.25%) is alone, without his or her nuclear family. The patriarchal figure is identified, in which women stay at home and men go to work to bring food and basic elements to their homes. The students highlight important aspects of their context through the drawings, mainly their relationship with the countryside (rural sector), dogs, cats, birds, chickens, cattle and fish. The Puracé volcano appears as a symbol of representation of the region and also highlights the daily chores in their homes, such as feeding the animals, putting up and fixing fences and housework (linked to the female sex). The recreational activity that is evident in some of the drawings is soccer, which is enjoyed with classmates from the same school. Only one drawing presents a character from the health sector and its author indicates that this is what he would like to be when he grows up. Figure 1 shows the work team with the members of the school. Figure 2 shows some of the children’s drawings and some of their paintings in the participatory diagnosis highlight important aspects of their context.

With the help of tablets from the Smart School program (Gonzalez Serrano 2015) and the Google Maps application (without an internet connection), the children identify their places of residence, the school and the routes they travel to travel from one place to the other. The results of this activity were not very favourable, as 10 children (62.5%) were not able to identify their homes or the routes they travel; however, six children (37.5%) did identify the location of their homes, having the school and the church as points of reference, but they did not determine the route they travelled. Although the application allows locating strategic points (school and church), the application does not present roads, routes or other key reference sites for children to locate their homes. The estimated time it takes children to go from their homes to school is between 10 min and 2 h, with the shortest time taken by those living in the village of Quintana and the longest by those living in the village of El Canelo. The means of transportation used are horses, motorcycles and the truck that transports milk. Many of the children walk to school.

In another session, a meeting was held with the parents of the children. It is important to clarify that the number of parents and attendees was 12, a group made up of seven women and five men. Of the group of parents attending, seven people (58.4%) are under 30 years old, seven people (58.4%) have more than one child attending school, and seven people (58.4%) live in the village of Quintana. Parents also indulged in the activity of identifying their homes and their routes to school using tablets and the Google Maps application (without an internet connection). This exercise was much simpler for the parents, who because of their age and knowledge of the region found it easier to locate themselves. In this way, reference points were established in space, for example, the church and the main road, to explain which roads their children use and the natural or social risks to which they are subject, and they also described the time it took for them to go one way or the other, depending on the means of transportation used.

### Regarding knowledge of environmental risks

Parents and guardians recognise that the most common incidents related to natural or man-made situations are: landslides, which occur in winter during the rainy season, and fires, which occur during the summer because of increased temperatures. These incidents are handled by the community itself and rarely with the help of the firefighters or organisations associated with risk management of the Popayán Municipal Mayor’s Office. Parents expressed the need to have an accompaniment that generates awareness about the most common incidents in the region, how to act and the mechanisms or procedures to request support from relief and disaster response agencies. Parents, guardians and the community in general state that they have neither the knowledge nor the necessary tools to act in the event of an emergency or disaster. Likewise, they state that the agencies almost never appear and are unaware of the existence of a contingency plan for all disasters that may occur.

On the other hand, the children, knowing their context, know what risks they are exposed to; however, they do not conceive them as a risk, but as natural phenomena that do not affect anyone. Half of the students recognise the sounds produced by some natural disasters. Among the best-known natural and man-made events are landslides, volcanic eruptions, forest fires and floods, which are easily recognised by older children. At the moment of identifying them in images, the children give reasons for them, but often not with their proper names but with the elements that produce them. Forest fires are catalogued as fire; volcanic eruption is ‘when the volcano spews fire’; as for the overflowing of rivers, they indicated that ‘it is caused by heavy rains and causes disasters in houses, drags people, animals and crops’. The children recognise that if a natural hazard occurs, they must go to the meeting point within the school and look for ways to ask for help from the police, firefighters and other agencies, which do not have a direct or permanent presence in the region. After determining the events, with the help of the working group, the children recognise that events can generate various problems in their school and community and that it is necessary to be prepared for any event that affects the safety of their school and their classmates; however, it is in the next phase of the project that they will talk about natural and man-made disasters and how to act in the face of them, considering an environmental risk-management plan. In the creation of stories related to natural disasters, the children selected the village of Quintana as the main scenario, reflecting that sense of belonging and concern for the place they live in.

### Regarding communication processes

Regarding the form of communication between parents and school, voice-to-voice and notes in the students’ notebooks are the most common, according to what parents said. The cell phone is also used; however, because of the lack of a cell phone signal or stable internet, communication is difficult. In this sense, parents and teachers stated that the existing means of communication are inefficient, because of the context in which they live and the distances they must travel to get to school and that the main need is for information and immediate communication among them. The preferred means of communication for parents to get information are radio and television as these are the media with the widest coverage and easiest access in rural areas. Television is generally watched in the morning before starting the workday (between 4:00 and 6:00), at midday and night.

Among the most tuned television (TV) channels are Caracol and RCN. Radio is listened to in the mornings through amplitude modulation (AM) stations such as Radio Súper 1070 and Caucana 1040, and frequency modulation (FM) stations such as Radio Uno. It should be noted that some community members have developed a form of inter-verbal communication through the use of radios (walkie-talkies), mainly from the ASOCAMPO organisation, which leads to productive, and communication projects. In relation to the children’s knowledge of means of communication, 100% know cell phone, 85.7% know TV and radio, 64.2% know computers, 50% know written media (books and letters), 42.8% know landlines and 7.1% know sign language and interpersonal communication. Regarding the means of communication in their homes, 100% said they had a cell phone, 85.7% had a television, 71.4% had a radio, 21.4% had access to written media (books or newspapers) and 21.4% said they had a computer. None of the boys or girls reported having a fixed telephone. This led to the conclusion that the means of communication most used by children’s families and by the community, in general, is the cell phone, perhaps because of its popularity, easy access and simplicity in establishing communication. The landline telephone is a non-existent medium in this population and access to written media and the use of tools such as computers and the internet are unlikely in the area.

Children, in general, are aware of the use of media and expressed the following functions: ‘watch movies, soap operas, and cartoons’, ‘the cell phone my dad uses it to call’, ‘the cell phone to call and send messages’, ‘the radio to listen to news’, ‘The radio to listen to music’, ‘the cell phone to play games’, ‘the cell phone to play and chat’, ‘send letters’, ‘tell stories’, ‘read’, ‘write’, ‘do homework’, which can be summarised in three functions: entertainment, information and communication. The television channels that are most watched by students are Canal Caracol, Canal 1, RCN and Señal Colombia. After identifying the media known and owned by fathers, mothers and children, we tried to reinforce the knowledge of their functions and to generate a process of awareness of their use, especially radio, as the medium to be promoted in this area to improve the information and communication processes between the school, families and the community in general.

## Discussion

It is necessary to emphasise that the rural context implies understanding other dynamics of life, teaching, learning and other needs. Although the facilitating team planned each session, several activities and tools were adapted to achieve the participation of the children, their parents and guardians and to achieve the proposed objectives. The participants reflected teamwork values during the different activities. Given that the children were part of a multi-grade class, the activities were adapted and distributed according to their age. In each meeting, the initial dynamics were of great importance, as they were activities that allowed for better interaction and confidence-building between the children and the facilitators. Likewise, at the end of the day, games and playful activities were conducted to allow them to return home happy.

The results evidenced that the school does not have an environmental risk-management plan or an action or contingency plan for natural disasters and associated catastrophes in the school. The children know the meeting point but there is a lack of knowledge about evacuation routes and other meeting points, and they do not know what to do in the event of a natural disaster. No public or private institution has conducted training on possible disasters. The community expresses its dissatisfaction because proposals arrive in the territory only during election times and are not implemented. The lack of tools to mitigate emergencies is notorious. Particularly, in the case of a volcano eruption, the community is very unconcerned about what could happen to them, as they have not had to live an experience of this type, and they consider that the noises of the earth and seismic movements are very natural. In this sense, the peasant and indigenous people have a different dimension or way to evaluate the risk, in comparison to the people of the city, and rural children consider every possible hazard as a threaten. Assessing and identifying the knowledge on prevention and response to environmental risks and natural disasters is fundamental for planning and designing educational programs for rural communities to face disasters. This initial approach to the community allows generating a feeling of confidence, encouraging the design, implementation and deploying of educational-communicative workshops regarding the problems and needs of the community.

Despite the reduced number of participants in the participatory diagnosis, the results are very conclusive, and they suggest that a participatory methodology is the best option to build an environmental risk-management plan and to look for the appropriated education and communication tools in the rural areas to get a safer school and to reduce the vulnerability of the communities. It is also important to count with a transdisciplinary facilitating team and to include in all the processes the community as the main actor, to ease the appropriation and sustainability of the project.

## Conclusions

This article presented the results of a participatory assessment of knowledge on the prevention and response to environmental hazards and natural disasters in the rural school Las Huacas, village of Quintana, Popayán, Cauca, Colombia:

- The research revealed that the most prevalent environmental threats, according to the context, are landslides, forest fires, volcanic eruptions, seismic movements, river overflows and floods. Other risks were identified in the children’s journey from home to school. Firstly, the long distances that some of them must travel, as well as the teacher and parents; secondly, the heavy rains and cold that are characteristic of the region and that affect the arrival at school and finally, crossing streams and dangerous waste and the presence of dogs and strangers.
- On the knowledge of environmental risks, fathers, mothers, guardians, boys and girls can identify the risks; however, they do not know how to act effectively and efficiently when facing them. The children identify the meeting point and aid and relief agencies (police and firefighters) to ask for help, but they do not know the correct way to act in the event of a natural disaster.
- Regarding communication processes, there is a lack of effective and immediate means of communication and tools between the teacher and the parents (and the community in general).
- The qualitative methodological approach was appropriate as it allowed a seamless interaction among the actors involved, generating a horizontal communication that allowed a process of dialogue and knowledge building according to each experience.
- The participation and the commitment of the community might allow in the next stages of the research project to look for an answer to the general research question on how to use and appropriate ICTs, the access to the information, the experience and the knowledge of the community, in the construction of mechanisms, tools and plans of environmental risk management in rural educative institutions, to benefit the children and their community.
